# Ectopic Expression of a Heterologous Glutaredoxin Enhances Drought Tolerance and Grain Yield in Field Grown Maize

**DOI:** 10.3390/ijms22105331

**Published:** 2021-05-19

**Authors:** Tej Man Tamang, Stuart A. Sprague, Tayebeh Kakeshpour, Sanzhen Liu, Frank F. White, Sunghun Park

**Affiliations:** 1 Department of Horticulture and Natural resources, Kansas State University, Manhattan, KS 66506, USA; tejman@ksu.edu (T.M.T.); SSPRAGUE@nwmissouri.edu (S.A.S.); tayebeh@ksu.edu (T.K.); 2 Department of Plant Pathology, Kansas State University, Manhattan, KS 66506, USA; liu3zhen@ksu.edu; 3 Department of Plant Pathology, University of Florida, Gainesville, FL 32611, USA; ffwhite@ufl.edu

**Keywords:** glutaredoxin S17, drought tolerance, grain yield, pollen

## Abstract

Drought stress is a major constraint in global maize production, causing almost 30–90% of the yield loss depending upon growth stage and the degree and duration of the stress. Here, we report that ectopic expression of *Arabidopsis glutaredoxin S17* (*AtGRXS17*) in field grown maize conferred tolerance to drought stress during the reproductive stage, which is the most drought sensitive stage for seed set and, consequently, grain yield. *AtGRXS17*-expressing maize lines displayed higher seed set in the field, resulting in 2-fold and 1.5-fold increase in yield in comparison to the non-transgenic plants when challenged with drought stress at the tasseling and silking/pollination stages, respectively. *AtGRXS17*-expressing lines showed higher relative water content, higher chlorophyll content, and less hydrogen peroxide accumulation than wild-type (WT) control plants under drought conditions. *AtGRXS17*-expressing lines also exhibited at least 2-fold more pollen germination than WT plants under drought stress. Compared to the transgenic maize, WT controls accumulated higher amount of proline, indicating that WT plants were more stressed over the same period. The results present a robust and simple strategy for meeting rising yield demands in maize under water limiting conditions.

## 1. Introduction

The world population is expected to grow to 9.7 billion by 2050, and global food production should be doubled to meet the demand [1,2]. Drought is one of the environmental stresses that affects the quality and quantity of crop production [3,4]. Drought severely affects plant growth and development and, consequently, leads to the death of the plant by affecting various physiological and biochemical processes such as photosynthesis, respiration, and nutrient uptake and translocation [5]. It is estimated that drought events will be more frequent, intense, and longer in the agriculturally important areas and pose a serious threat to the food security [3,6,7]. Since 1990, drought has affected almost 2 billion people and has cost the global economy losses of USD 6 to 8 billion annually [7]. Drought impacts maize production and growth from vegetative stages to the reproductive stages [8]. The 2012 US drought caused a substantial impact in the global agricultural market [9,10]. During this drought period, corn production declined by more than 25% [10]. Similarly, the 1988 drought in the Midwest region caused a 30% decrease in corn production and cost USD 3 billion in relief payments [11].

Transgenic approaches have been made to improve drought tolerance in plants. These include engineering of the genes that encode proteins related to osmotic adjustment, plant hormone synthesis, ion transporters, chaperones, transcription factors, and reactive oxygen species (ROS) [12,13,14]. In maize, overexpression of *ZmZAR1* improved plant growth and traits related to drought-stress tolerance [15]. Ectopic expression of *Arabidopsis NF-YB2* in maize exhibited higher chlorophyll index, photosynthesis rate, stomatal conductance, and 50% more yield in field [16]. Similarly, RNAi lines created by knocking down the *ACC synthase* in maize showed reduced anthesis-silking interval, thereby, increasing kernel set [17]. Drought tolerance in maize has also been reported to be improved by ectopically expressing genes such as *NPK1* from tobacco, *TsCBF1* and *TsVP* from *Thellungiella halophila, betA* from *E.coli*, *HVA1* from Barley, and *TPP* from rice [18,19,20,21,22,23].

ROS function as signaling molecules under the control of antioxidant system [24]. However, under abiotic and biotic stresses, including drought, ROS production is rapidly increased. Overproduction of the ROS disables the antioxidant defense system and causes oxidative damage to the lipids, proteins and DNA [25]. Glutaredoxins (GRXs) are small ubiquitous oxidoreductase that belongs to thioredoxin family. These group of proteins maintain the cellular ROS homeostasis by catalyzing the reduction of disulfide bonds of target proteins through dithiol or monothiol mechanism in the presence of glutathione [26]. Glutaredoxins are predicted to be found in various parts of the cells such as cytosol, chloroplast, and mitochondria and play a role in the oxidative stress response [27]. They are also involved in Fe-S cluster assembly which is required for several processes, including photosynthesis, respiration, nitrogen and sulfur assimilation, ribosome synthesis and DNA repair [27]. Based on the active site motifs, plant GRXs are grouped in four classes. Class I, class II, class III, and class IV have CxxC/S, CGFS, CCxx, and CxDC/s or CPxC active site motifs, respectively [28]. *Arabidopsis AtGRXS17* is a member of the class II GRXs that was shown to be involved in maintaining the ROS accumulation, auxin signaling, and temperature-dependent postembryonic growth in the plants [29]. Ectopic expression of *AtGRXS17* in plants has been reported to improve the plant tolerance against several abiotic stresses, such as heat, chilling, and drought, at the vegetative stages [30,31,32]. The expression of the *AtGRXS17* is also crucial for maintaining the ROS homeostasis in plants and, thus, protects the plants from oxidative damage [31,33]. However, the effect of *AtGRXS17* in response to drought stress during the reproductive stage, particularly in the field, has not been studied yet. 

Maize is one of the most important crops in the world and is widely used for food, feed, and biofuel. More than 1100 million metric tons of maize were produced worldwide, and over 30% of the maize was produced in the U.S., which is the largest producer and exporter of maize [34]. Here, we investigate the effects of *AtGRXS17* in field-grown maize on the drought tolerance during the reproductive growth stages and demonstrate that ectopic expression of this single gene in maize can increase the grain yield under drought stress conditions.

## 2. Results

### 2.1. Upregulation of ZmGRXS17 Expression under Drought Stress

Maize endogenous monothiol glutaredoxin S17, *ZmGRXS17*, expression was measured in leaf tissue of various inbred lines (B73, B104, A188, and HiIIA) in response to drought stress. The expression pattern of *ZmGRXS17* under drought stress was similar across the inbred lines (Supplementary Figure S1a). *ZmGRXS17* transcripts increased in response to the drought on day 2 and decreased on day 4 of drought stress in all the inbred lines. However, the *ZmGRXS17* expression increased gradually after 4 days as the period of drought was extended. In the maize line B104, the transcript expression was 5.9 times higher than the initial levels after 2 days of drought stress. The relative expression of *ZmGRXS17* was 2.2 times on day 4, 3.6 times on day 6, and 4 times higher on day 8 of the drought stress compared to the initial level of expression (Supplementary Figure S1a).

### 2.2. AtGRXS17-Expressing Maize Plants Increase Yield under Drought Stress Field Conditions

**Figure 1:**
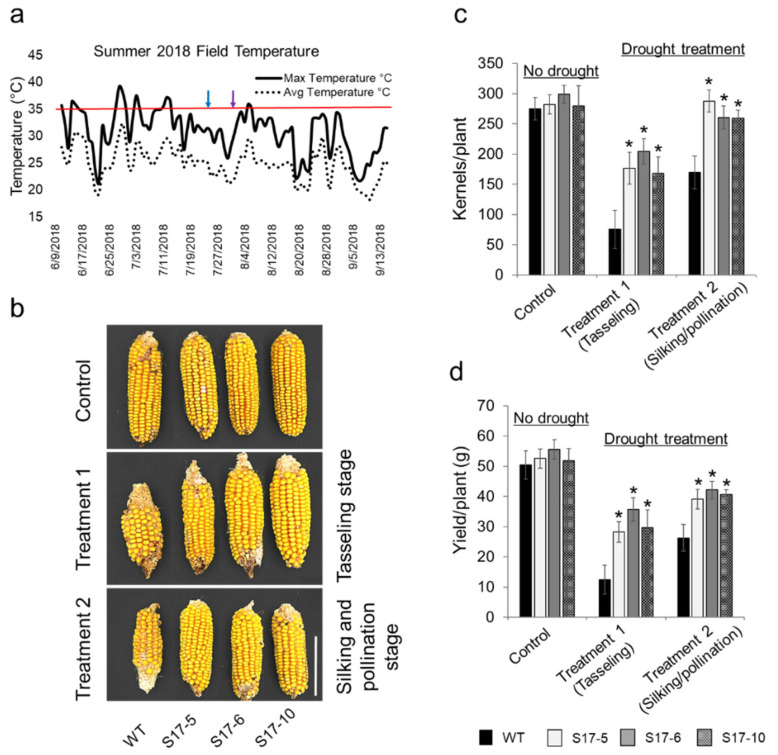
Effect of ectopic expression of *AtGRXS17* in maize yield under drought in the field. (**a**) Average and maximum daily temperature during the drought stress. The blue arrow represents the drought stress initiation at the VT stage and purple arrow represents the drought initiation at the R1 stage. During the period of the drought stress, the maximum temperature remained below 35 °C as represented by a red bar; (**b**) Cobs from WT and *AtGRXS17*-expressing lines grown in the field under controlled condition and drought condition at VT (Tasseling stage) and R1 (Silking stage). Scale bar = 10 cm; (**c**) Average yield per maize plant harvested from field grown maize at well-watered condition (*n* = 12) and drought at the VT (*n* = 12) and R1 (*n* = 12) stages; (**d**) Number of kernels per plant grown in the field under well-watered condition and drought at the VT and R1 stages. Data shown are Mean ± SE and were analyzed using one-way ANOVA. Asterisks indicate significance level (* *p* < 0.05).

*AtGRXS17*-expressing maize lines (*S17*-*5*, *S17*-*6*, and *S17*-*10*) along with wild-type (WT) B104 plants were grown in the field. Drought stress was imposed at VT (tassel) and R1 (silking) stages by withholding watering, and the yields were compared. During the period of drought stress, the average maximum daily temperature and average daily temperature were 30.4 °C and 23.5 °C, respectively (Figure 1a). Drought stress had less impact on the kernel number set in each ear of *AtGRXS17*-expressing lines compared to the WT plants at both VT and R1 stages (Figure 1b). Kernel numbers under drought treatments at the VT stage were 76, 176, 204, and 168 for WT, *S17*-*5*, *S17*-*6*, and *S17*-*10*, respectively. Similarly, kernel numbers under the drought treatments at the R1 stage were 170, 288, 260, and 259 for WT, *S17*-5, *S17*-6, and *S17*-10, respectively (Figure 1c). In WT and transgenic maize, the weight of seeds per thousand was not different in control and drought during the R1 stage. However, when drought stress was imposed at the VT stage, *AtGRXS17*-10 had significantly higher seed weight compared to the WT and other *AtGRXS17*-expressing lines (Supplementary Figure S1b). On average, *AtGRXS17*-expressing lines showed at least 127% and 48% more yield than WT plants when drought stress was imposed at VT and R1 stage in the field, respectively (Figure 1d). No significant difference in the yield was found between *AtGRXS17*-expressing lines and WT plants under well-watered conditions (Figure 1b–d). Similar results were observed in the greenhouse experiments. All the *AtGRXS17*-expressing lines had a higher yield than WT plants when drought stress was imposed at VT stage in the greenhouse (Supplementary Figure S2a,b). Our data also indicated that the drought stress was more severe if imposed at the VT stage when compared with the R1 stage in the field (Supplementary Figure S2c).

### 2.3. AtGRXS17-Expressing Maize Pollen Is More Drought Tolerant Than WT Pollen

**Figure 2:**
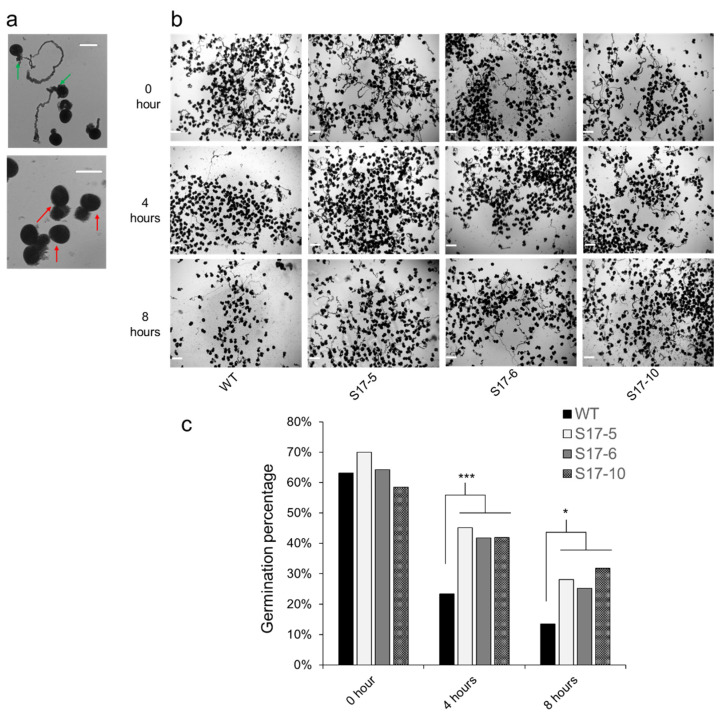
Pollen germination of the *AtGRXS17*-expressing and WT maize under drought stress. (**a**) Pollen germination (Upper panel; green arrow) and pollen that failed to germinate (Lower panel; red arrow) in the pollen germination media; (**b**) Time course germination rate of pollen kept at room temperature; (**c**) Pollen germination rate at 0, 4, and 8 h timepoint of the WT and *AtGRXS17*-expressing maize plants. Scale bars = 100 µm. Data shown are Mean ± SE (*n* = 3) and were analyzed using one-way ANOVA. Asterisks indicate significance level (* *p* < 0.05, *** *p* < 0.001).

Pollen grains were collected at 0, 4, and 8 h and incubated in the pollen germination media. A total of 15,176 pollen grains were scored for the germination, which was based on the pollen tube that was at least equal to the diameter of the pollen grain (Figure 2a). Fresh pollen collected from all tassels at 0 h had an average of 64% germination rate. Pollen germination rate significantly reduced in WT after 4 h of desiccation period. After 4 h, WT pollen germination was 23.4% and *AtGRXS17*-expressing pollen was 42.9%. After 8 h, the pollen germination rate was 28.3%, more than double in *AtGRXS17*-expressing pollen compared to WT pollen (Figure 2b,c).

### 2.4. Effect of Ectopic Expression of AtGRXS17 on Proline Accumulation, Chlorophyll Content, Relative Water Content, Stomatal Conductance, and H_2_O_2_ under Drought Conditions

**Figure 3:**
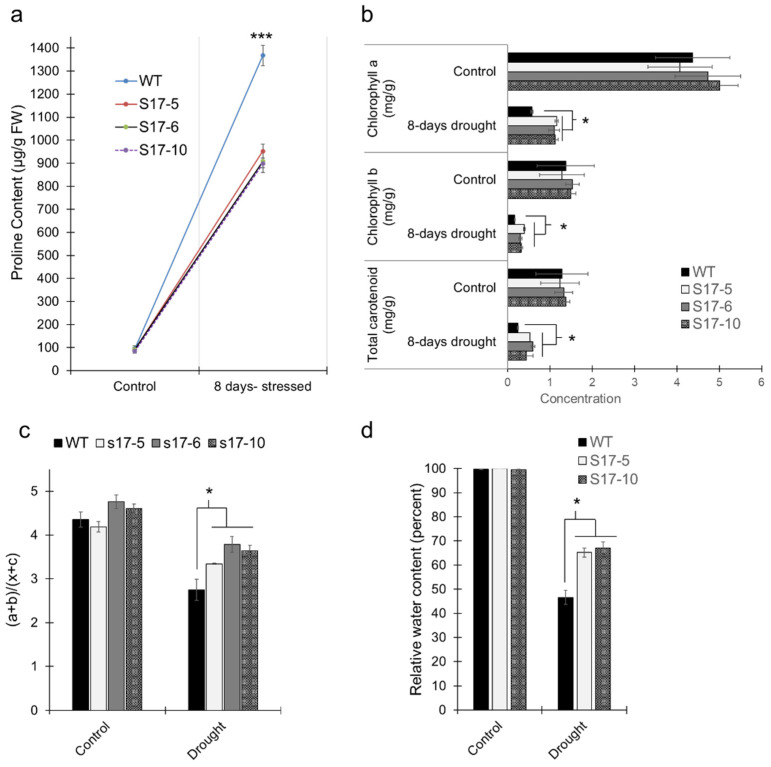
*AtGRXS17*-expressing maize plants were less stressed than WT plants under drought stress. (**a**) Proline accumulation of WT and *AtGRXS17*-expressing maize plants under control (well-watered) and drought stress after 8 days; (**b**) Total chlorophyll and carotenoid content in the WT and *AtGRXS17*-expressing maize plants under control (well-watered) and drought stress after 8 days; (**c**) Graph represents the stress as measured by the ratio of total chlorophyll to total carotenoids; (**d**) Relative water content of 4-week-old WT and two *AtGRXS17*-expressing maize seedlings under control (well-watered) and drought stress after 5 days. Data shown are Mean ± SE (*n* = 6) and were analyzed using one-way ANOVA. Asterisks indicate significance level (* *p* < 0.05, *** *p* < 0.001).

Proline content was not different between WT and *AtGRXS17*-expressing maize plants under control conditions. After 8 days of drought treatment, the proline content increased in both WT and *AtGRXS17*-expressing maize plants. However, the proline content of WT plants was higher than that of transgenic plants (Figure 3a). Chlorophyll (a and b) and carotenoid content of the transgenics and WT plants were not different under the well-watered conditions, while the *AtGRXS17*-expressing plants had higher concentrations of chlorophyll and carotenoids than the WT plants under drought conditions (Figure 3b). Similarly, the ratio of chlorophyll to carotenoid content was also higher in *AtGRXS17*-expressing maize plants under drought conditions (Figure 3c). Relative water content (RWC) declined in both *AtGRXS17*-expressing and WT maize plants after 5 days of drought treatment. However, transgenic maize plants retained more water than the WT plants. The RWC in WT dropped to 46.6%, while RWC in transgenic maize plants dropped to 66% (Figure 3d). 

**Figure 4:**
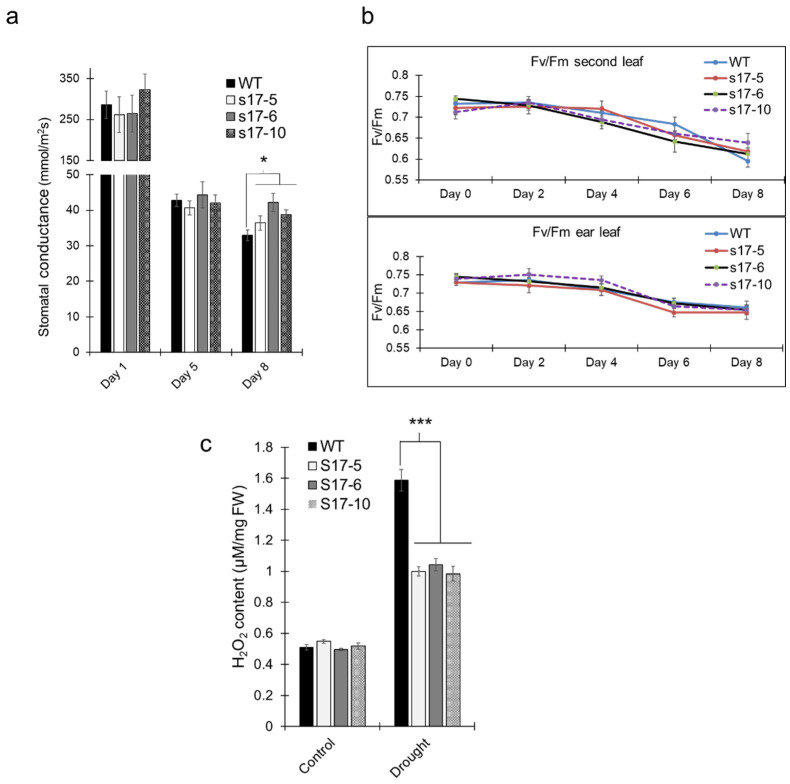
Effect of ectopic expression of *AtGRXS17* in stomatal conductance and H_2_O_2_ content. (**a**) Stomatal conductance of WT and *AtGRXS17*-expressing maize plants at VT stage as the drought progresses; (**b**) Chlorophyll fluorescence measurement of the ear leaf and second leaf from top in WT and *AtGRXS17*-expressing maize plants at VT stage as the drought progresses; (**c**) H_2_O_2_ content of the WT and *AtGRXS17*-expressing maize plants under control (well-watered) and drought stress after 8 days. Data shown are Mean ± SE (*n* = 6) and were analyzed using one-way ANOVA. Asterisks indicate significance level (* *p* < 0.05, *** *p* < 0.001).

Stomatal conductance of both *AtGRXS17*-expressing and WT plants gradually decreased as drought stress was imposed. Stomatal conductance remained similar for both the transgenic and WT lines on day 1 and day 5 of drought treatments but decreased in WT plants on day 8, while remaining steady in *AtGRXS17*-expressing plants (Figure 4a). Chlorophyll fluorescence, as measured by Fv/Fm ratio, gradually decreased under drought treatment. No difference in the chlorophyll fluorescence was observed on ear leaf and second leaf from top in both WT and *AtGRXS17*-expressing lines over the drought period (Figure 4b). H_2_O_2_ content was similar in both WT and *AtGRXS17*-expressing maize plants when grown in well-watered conditions. H_2_O_2_ significantly accumulated in WT plants when compared with *AtGRXS17*-expressing maize plants under drought conditions (Figure 4c).

### 2.5. Drought Stress Results in Altered Gene Expression in Maize Plants

**Figure 5:**
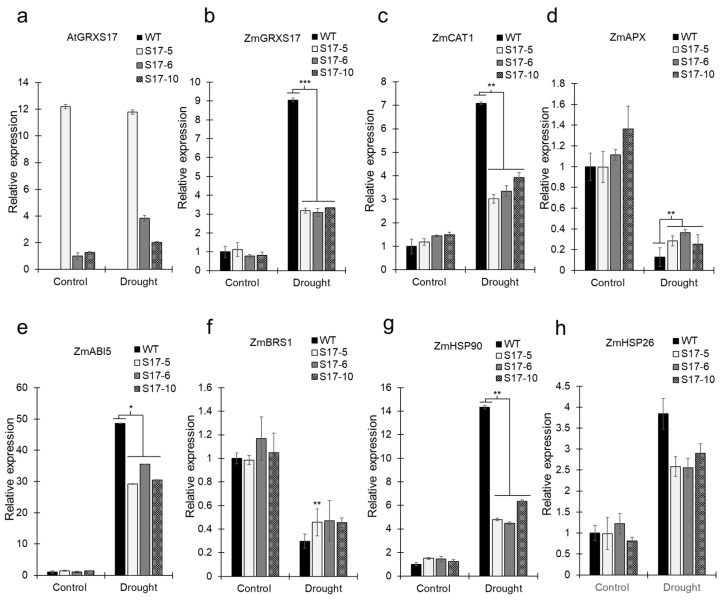
A relative comparison of transcript levels between *AtGRXS17*-expressing and WT maize plants in response to drought stress; (**a**) *AtGRXS17*; (**b**) *ZmGRXS17*; (**c**) Catalase (*ZmCAT1*); (**d**) L-ascorbate peroxidase (*ZmAPX*); (**e**) ABSCISIC ACID-INSENSITIVE 5 (*ZmABI5*); (**f**) Brassinosteroid synthesis 1 (*ZmBRS1*); (**g**) Heat shock protein, 90 KDa (*ZmHSP90*); (**h**) Heat Shock protein, 26 KDa (*ZmHSP26)*. Data were analyzed using one-way ANOVA. Asterisks indicate significance level (* *p* < 0.05, ** *p* < 0.01, *** *p* < 0.001).

Drought treatments induced changes in expression of drought-stress related genes (Figure 5a–h). Though yield difference among the transgenic lines was not observed, *S17-5* displayed the highest expression of the transgene *AtGRXS17*, followed by *S17*-*6*, and *S17*-*10* (Figure 5a). *ZmGRXS17*, an endogenous *GRXS17* of maize, had an increase in the expression in WT plants in comparison to the *AtGRXS17*-expressing maize lines under drought conditions (Figure 5b). Similarly, antioxidation enzymes showed varied effect of drought stress on their expression. Under drought, *catalase* (*ZmCAT1)* expression increased in WT plants whereas *L-ascorbaste peroxidase* (*ZmAPX*) expression was lower in WT plants when compared to *AtGRXS17*-expressing maize lines (Figure 5c,d). *ZmABI5*, *ABSCISIC ACID-INSENSITIVE 5*, expression was higher in the WT plants than *AtGRXS17*-expressing maize lines after drought treatment (Figure 5e). *Brassinosteroid synthesis 1* (*ZmBRS1*) expression decreased under drought conditions, less in WT plants than *AtGRXS17*-expressing lines (Figure 5f). *Heat shock proteins* (*HSPs*) experienced higher expression of both *ZmHSPs* (*ZmHSP90* and *ZmHSP26*) in WT plants than *AtGRXS17*-expressing maize lines after drought treatment (Figure 5g,h).

## 3. Discussion

Maize is a drought-sensitive crop, and water-deficit conditions during the pollination and early grain-filling stages, specifically, cause yield loss [35]. Similarly, drought imposition at the VT stage was found to have greater negative impact in corn grain yield than imposition at the vegetative stage [36]. Drought increases ROS production, which can affect cellular processes by causing oxidative damage. Here, drought stress imposition at the VT stage also had negative yield impact, and ectopic expression of *AtGRXS17* in maize reduced H_2_O_2_ accumulation under drought stress, indicating that *AtGRXS17* plays a role in reducing ROS accumulation during drought stress. We observed that endogenous *ZmGRXS17* was upregulated during drought stress in both *AtGRXS17*-expressing and WT lines (Figure 5b). We also found that *ZmGRXS17* expression was lower in *AtGRXS17*-expressing lines than in WT lines, which leads us to suggest the possibility that *AtGRXS17* expression may compensate the functional activity of endogenous *ZmGRXS17* in transgenic maize lines.

Pollen viability can be affected by several factors, including moisture. Maize pollen is considered “desiccation intolerant” and loses water and viability rapidly [37,38]. B104 pollen viability is lost when pollen moisture content is around 32% [39]. We found that pollen viability rapidly decreased even more when pollen grains were kept for a longer period in a desiccating environment. *AtGRXS17*-expressing plants, on the other hand, maintained higher pollen viability compared to WT plants after 2 and 4 h of desiccation. Furthermore, WT pollen viability went below 15% after 8 h, while *AtGRXS17*-expressing maize pollen viability only dropped to 28%, indicating that the higher kernel set in the *AtGRXS17*-expressing plants can be achieved in comparison to WT plants unless extreme conditions.

*AtGRXS17*-expressing maize lines have higher stomatal conductance, RWC, higher chlorophyll levels, and carotenoid contents than WT plants under drought stress. Drought stress and low water content generally lead to the stomatal closure, resulting in decreased transpiration and photosynthesis [40]. This, in turn, increases ROS accumulation that can cause heavy damage to the cells if remained unchecked [41,42]. A high RWC is a drought resistance mechanism rather than the drought-escape mechanism [36,43]. *AtGRXS17*-expressing plants were similar to drought tolerant genotypes BC678 and BC404, which have higher chlorophyll contents and showed higher yield due to less damaged chloroplast [44]. Similarly, drought tolerant cultivars of wheat had higher RWC and chlorophyll content [45]. The ratio of weight of chlorophyll to carotenoid was low in the WT plants under drought stress, which is an indicator of plant stress and damage to the photosynthetic apparatus [46]. By these indicators, *AtGRXS17*-expressing maize plants were less stressed than WT plants. Furthermore, after 8 days of drought stress, WT plants accumulated approximately 32% more proline than *AtGRXS17*-expressing maize lines, indicating, again, that the WT plants were more stressed under drought conditions as compared to the *AtGRXS17*-expressing plants. Wild-type plants also accumulated approximately 36% more H_2_O_2_ than *AtGRXS17*-expressing maize lines. Proline accumulation increases when plants get stressed, and the accumulation is dependent upon H_2_O_2_ accumulation. The higher proline content could be an indicator of the degree of drought stress in the maize plants. Proline accumulation could be due to reduced water status in the maize plants, rather than the drought resistance mechanism [47]. We postulate that *AtGRXS17* expression assisted maize plants to maintain water status during drought which, in turn, caused the transgenic plants to accumulate less H_2_O_2_ and proline, subsequently. 

H_2_O_2_ promotes proline accumulation by way of abscisic acid (ABA) in Arabidopsis [48]. ABA acts as a key regulator for abiotic stress resistance in plants [49]. Endogenous ABA increases under drought stress in maize [50,51] and participates in a network of stress-signaling mechanisms [52]. Exogenous application of ABA can improve the drought tolerance in various plant species [53,54]. Exogenous application of ABA is also known to enhance the activities of antioxidant enzymes, such as catalase, peroxidase, ascorbate peroxidase, superoxide dismutase, and glutathione reductase [55]. We observed that ABA INSENSITIVE 5 (ABI5), a leucine zipper transcription factor, expression increased by more than 30-fold under drought stress in both WT and AtGRXS17-expressing maize plants but relatively higher in WT plants as compared to AtGRXS17-expressing maize plants under drought stress. ABI5 is regulated by ABA [56,57] and functions in various abiotic stress adaptations [58]. Ectopic expression of ZmABI5 in tobacco showed that transgenic tobacco lines were more sensitive to drought, salt, high and low temperature, suggesting that ZmABI5 may play a negative regulatory role in stress response [59]. Transgenic Arabidopsis plants expressing OsABI5 were also sensitive to salinity and drought stress, indicating negative regulatory role in stress response [60].

We observed a novel expression pattern of *ZmCAT1* in our study under drought stress. *ZmCAT1* expression was greater in WT plants compared to *AtGRXS17*-expressing maize plants, contradicting studies involving heat, chilling, and drought stress where CAT activity increased in *AtGRXS17*-expressing tomatoes [30,31,32]. One possible explanation is that the higher *CAT1* expression in WT maize plants may be due to higher H_2_O_2_ accumulation in WT maize brought on by drought stress. Further work is required to understand different *CAT1* expression responses between *AtGRXS17*-expressing tomato and *AtGRXS17*-expressing maize under drought stress. Conversely, *ZmAPX* expression decreased by more than half under drought stress in both transgenic maize and WT plants. APX activity is shown to decrease in several plant species such as sorghum, rice, and maize under drought stress [61,62,63]. Our result showed the higher expression of *ZmAPX* in transgenic maize compared to WT plants under drought. *ZmAPX* expression at relatively higher levels might compensate to detoxify ROS in transgenic maize. A balance of ROS scavenging enzymes is crucial for ROS level suppression. When CAT activity decreases, activity of scavenging enzymes such as APX should increase as a compensatory mechanism [64].

Heat shock proteins (HSPs) are also targets of GRXs [65]. HSPs, termed as molecular chaperones, stabilize proteins at intermediate stages of their formation and assist in folding, association, translocation, and degradation of the proteins across the membranes [66]. HSPs are induced by several abiotic stresses, including drought stress [67]. However, expression of several *HSPs* has conferred sensitivity to abiotic stresses. In *Arabidopsis*, overexpression of *AtHsp90* conferred tolerance to high calcium, but plants were more sensitive to salinity and drought stresses [68]. Similarly, ectopic expression of the creeping bentgrass *AsHSP26.8a* in *Arabidopsis* resulted in the hypersensitivity to ABA and salinity stress and reduced tolerance to the heat stress [69]. Expression of *ZmHsp90* and *ZmHsp26* was relatively higher in WT plants as compared to *AtGRXS17*-expressing maize plants under drought stress. We suggest that WT plants could require higher expression of *HSPs* due to greater sensitivity to the drought stress than transgenic maize plants. Though HSPs might be interacting target proteins of GRXs, how AtGRXS17 interacts with HSPs is unknown. In the recent study, GRXS17 is found to possess both foldase and redox-dependent holdase activity which are further supporting the role of GRXS17 as a molecular chaperone [70].

*AtGRXS17*-expressing maize plants were less stressed under condition of drought stress in comparison to WT plants. The transgenic lines had higher RWC than the WT controls. We hypothesized that, in WT plants, stomata close earlier than in *AtGRXS17*-expressing lines under drought stress due to reduced plant water status. The reduced water status leads to higher accumulation of ROS in WT control plants as compared to *AtGRXS17*-expressing maize plants. The evidence, along with the higher pollen viability in *AtGRXS17*-expressing maize plants under drought stress, indicates that ectopic expression of *AtGRXS17* in maize confers tolerance to drought stress during the reproductive stage and improves grain yield.

## 4. Materials and Methods

### 4.1. Cloning AtGRXS17 and Plant Transformation

*AtGRXS17*-expressing, driven by maize *ubiquitin-1* (*Ubi-1*), B104 maize lines and subsequently homozygous *AtGRXS17*-expressing T2 lines were established previously [71]. Three lines, *AtGRXS17-5*, *AtGRXS17-6,* and *AtGRXS17-10* were used. Based on previous studies, *AtGRXS17-5* and *AtGRXS17-10* had one stable integration of T-DNA and *AtGRXS17-6* had multiple integrations of T-DNA into the genome. The phenotypes of transgenic and WT plants were indistinguishable under well-watered conditions [71]. Control plants (WT) had not transformed with vector or generated through tissue culture.

### 4.2. qRT-PCR Analysis of the Maize Endogenous Monothiol Glutaredoxin 17, ZmGRXS17

Maize inbred lines B73, B104, A188, and HiIIA were grown in PRO-MIX^®^BRK MYCORRHIZAE^™^ (Hummert International, St. Louis, MO, USA) at 27 °C/22 °C (day/night) in the greenhouse. At V5 growth stage, the seedlings were assigned to the drought treatments with three biological replicates. Plants were imposed with drought stress by withholding water. Leaf samples were collected from each plant on day 0, 2, 4, 6, and 8, and immediately frozen in liquid nitrogen and stored in −80°C freezer for further analysis.

Total RNA was isolated from leaf tissues of inbred maize lines using the Direct-zol RNA Miniprep Plus Kits (Zymo Research, Irvine, CA, USA). One µg total RNA was used to synthesize first strand cDNA using Revert Aid First Strand cDNA synthesis kit (Thermo Scientific^™^, Waltham, MA, USA). Each 20 µL qRT-PCR reaction consisted of 9.4 μL diluted cDNA solution (2 ng/μL), 0.3 μL of each primer (20 μM), and 10 μL iQ SYBR Green Supermix (Bio-Rad, Hercules, CA, USA). Primers 5′-CCGTCATCGCCTCACGAAGAG-3′ and 5′-AGAGCCTGCCTTACGGAATTGG-3′, which are based on the cyclin-dependent kinase gene CDK, were used as an expression control.

### 4.3. Field Trial

WT B104 and transgenic plants were grown in a randomized block design with 10 replications at the Rockyford Research station in Manhattan, KS. Seeds were germinated in 4-inch pots. Three weeks old seedlings were transferred into the 5-gallon pots containing PRO-MIX^®^BRK MYCORRHIZAE^™^ and pasteurized field soil in 1:1 ratio, and subsequently, transferred to the field. Approximately at V10 stage, poly film cover was placed on the rainout shelter to prevent the effect of rainwater during drought treatment. The first drought treatment that started at VT stage was continued for 6 days. The second drought treatment was started at R1 stage and continued for 5 days. After drought stress, the plants were watered regularly until harvest.

### 4.4. Greenhouse Experiment

WT and *AtGRXS17*-expressing lines were grown in the greenhouse at 27 °C/22 °C (day/night) in 1-gallon pots filled with equal volume of Pro-Mix^®^BRK Mycorrhizae^™^ until VT stage. At VT stage, drought stress was imposed by withholding water for 5 days. After 5 days, watering was restarted to recover, and pollination was performed manually under well-watered condition. After the drought stress, the plants were watered regularly until harvest.

### 4.5. Pollen Germination Analysis

Pollen grains from *AtGRXS17*-expressing lines and WT plants were collected at VT stage. Pollen was incubated on a liquid media comprising of 0.0005% boric acid, 10 mM calcium chloride, 0.05 mM monopotassium phosphate, 10% sucrose, and 6% polyethylene glycol 4000 for 30 min and observed under the light microscope. The tassel was kept in the room temperature inside a Petri dish to mimic a drought environment. The pollen germination was observed at 0, 4, and 8 h. In the in vitro study, germination was scored when the pollen tube length is at least equal to the diameter of the pollen grains. 

### 4.6. Proline Content Measurement

B104 and three *AtGRXS147*-expressing lines were grown until VT stage. The plants were grown under control and drought (8 days) conditions. Proline content was determined using a colorimetric assay as described previously [72]. Briefly, 100 mg of ground leaf samples were suspended into 500 µL of 3% sulfosalicylic acid and centrifuged for 5 min at 13,000× *g* rpm. Then, 100 µL of supernatant was added to 500 µL of reaction mixture that contains 100 µL of 3% sulfosalicylic acid, 200 µL of glacial acetic acid, and 200 µL of acidic ninhydrin solution. Then, the tubes were incubated at 96 °C for 1 h and the reaction was terminated on ice. 1 mL of toluene was added to the reaction mixture and the samples were vortexed for 20 s. The reaction was left at room temperature for 5 min to allow the separation of the organic and water phases. The upper organic phase was used to determine proline content by measuring the absorbance at 520 nm using toluene as reference. The proline concentration was determined using a standard concentration curve and calculated on FWbasis (µg/g).

### 4.7. Chlorophyll Content Measurement

WT along with three transgenic lines were grown in the greenhouse under optimum condition. Drought stress was imposed on maize plants at VT stage for 8 days. Chlorophyll content (chlorophyll a and b) along with the carotenoid contents were measured from control and drought-stressed plants as previously explained [73]. Briefly, chlorophyll contents along with carotenoids were extracted from 0.2 g of leaf samples using 100% acetone. The extract was centrifuged for 5 min at 500× *g*. Chlorophyll and carotenoid contents were determined by measuring the absorbance at 662 nm, 645 nm and 470 nm. The following equations were used to determine the chlorophyll and carotenoid contents.

Chlorophyll content: C_a_ = 12.25 A662 − 2.79 A645

Cb = 21.50 A645 − 5.10 A662

Total carotenoids (x + c) = (1000 × A470 − 1.82 × Ca − 85.02Cb)/198

The weight ratio of Chls a and b to the total carotenoid, (a + b)/(x + c), was determined.

### 4.8. Relative Water Content (RWC)

WT along with two *AtGRXS17*-expressing lines with one insertion were grown at 27 °C/22 °C (day/night) with 14-h photoperiod in the growth chamber. After 4 weeks, the seedlings were subjected to the drought treatment by withholding water for 5 days. The measurement of relative water content was performed as described previously [74]. Briefly, the leaves (2 cm × 3 cm) from control and drought-stressed seedlings were excised, and fresh weight (FW) was measured. Then, the leaves were immersed into the distilled water for 4 h and turgid weight (TW) was measured. Finally, dry weight (DW) was measured after drying the leaves at 80 °C overnight. The RWC was calculated as follows: RWC = (FW − DW)/(TW − DW).

### 4.9. Stomatal Conductance and Chlorophyll Fluorescence Measurement

WT and *AtGRXS17*-expressing lines were grown until VT stage. The plants were subjected to the drought. Stomatal conductance was measured using SC-1 leaf porometer (Decagon Devices, Pullman, WA, USA) on day 1, day 5, and day 8 of the drought treatment. Stomatal conductance was measured in the abaxial surface of the flag leaves following a 30-min dark adaptation. Similarly, phytochemical efficiency of the leaves was determined by chlorophyll fluorescence ratios (Fv/Fm) using portable chlorophyll fluorometer (OS30p+, Opti-Sciences, Hudson, NH, USA). Chlorophyll fluorescence measurement was taken from dark-adapted leaves on day 0, day 2, day 4, day 6, and day 8 of drought treatment.

### 4.10. H_2_O_2_ Assay

Hydrogen peroxide was measured from WT plants and *AtGRXS17*-expressing maize plants grown in controlled condition and drought-stressed condition using Amplex^®^ Red Hydrogen Peroxide/Peroxidase Assay kit (Invitrogen, Waltham, MA, USA). Briefly, 100 mg of ground leaf samples were suspended in 1× reaction buffer and mixed well. 50 µL of the working solution of 100 µM of Amplex^®^ Red reagent and 0.2 U/mL Horseradish Peroxidase was mixed to 50 µL of the sample solution in a well of a microplate and the reaction was incubated at the room temperature in dark for 30 min. The H_2_O_2_ concentration was determined by measuring the absorbance at 560 nm using no-H_2_O_2_ as control. H_2_O_2_ concentration was determined using a standard concentration curve and calculated on FWbasis (µg·mg^−1^).

### 4.11. qPCR of Leaf Tissues

WT and *AtGRXS17*-expressing lines were grown until VT stage. Flag leaf tissues from three biological replicates of WT and *AtGRXS17*-expressing lines grown in the well-watered condition and drought-stressed condition (8 days) were collected. Total RNA was isolated from leaf tissues of inbred maize lines using the Direct-zol RNA Miniprep Plus Kits (Zymo Research, Irvine, CA, USA). One µg total RNA was used to synthesize first strand cDNA using Revert Aid First Strand cDNA synthesis kit (Thermo Scientific^™^, Waltham, MA, USA). qRT-PCR was carried for different genes listed in the Supplementary Table S1.

### 4.12. Statistical Analysis

Data were analyzed using one-way analysis of variance (ANOVA). The data presented are the means ± standard error (SE) of at least six biological replicates for WT and *AtGRXS17*-expressing lines. Statistical significance was attained at a probability level of *p* < 0.05.

## Data Availability

Not applicable.

## Supplementary Materials

The following are available online at https://www.mdpi.com/article/10.3390/ijms22105331/s1, Figure S1: Expression levels of *ZmGRXS17* in inbred lines. Figure S2: Effect of *AtGRXS17* expression in maize yield under drought stress. Table S1: Primers used for qRT-PCR.

## References

[1] Tilman D., Balzer C., Hill J., Befort B.L. (2011). Global Food Demand and the Sustainable Intensification of Agriculture. Proc. Natl. Acad. Sci. USA.

[2] United Nations, Department of Economic and Social Affairs, Population Division World Population Prospects Highlights; 2019; ISBN 978-92-1-148316-1. https://population.un.org/wpp/Publications/Files/WPP2019_Highlights.pdf.

[3] Lesk C., Rowhani P., Ramankutty N. (2016). Influence of Extreme Weather Disasters on Global Crop Production. Nature.

[4] Mishra V., Cherkauer K.A. (2010). Retrospective Droughts in the Crop Growing Season: Implications to Corn and Soybean Yield in the Midwestern United States. Agric. Forest Meteorol..

[5] Jaleel C.A., Manivannan P., Wahid A., Farooq M., Al-Juburi J., Somasundaram R., Panneerselvam R. (2009). Drought Stress in Plants: A Review on Morphological Characteristics and Pigments Composition. Int. J. Agric. Biol..

[6] Xu H., Twine T.E., Girvetz E. (2016). Climate Change and Maize Yield in Iowa. PLoS ONE.

[7] Zipper S.C., Qiu J., Kucharik C.J. (2016). Drought Effects on US Maize and Soybean Production: Spatiotemporal Patterns and Historical Changes. Environ. Res. Lett..

[8] Zheng J., Fu J., Gou M., Huai J., Liu Y., Jian M., Huang Q., Guo X., Dong Z., Wang H. (2010). Genome-Wide Transcriptome Analysis of Two Maize Inbred Lines under Drought Stress. Plant Mol. Biol..

[9] Al-Kaisi M.M., Elmore R.W., Guzman J.G., Hanna H.M., Hart C.E., Helmers M.J., Hodgson E.W., Lenssen A.W., Mallarino A.P., Robertson A.E. (2013). Drought Impact on Crop Production and the Soil Environment: 2012 Experiences from Iowa. J. Soil Water Conserv..

[10] Rippey B.R. (2015). The U.S. Drought of 2012. Weather Clim. Extrem..

[11] Rosenzweig C., Iglesius A., Yang X.B., Epstein P.R., Chivian E. (2001). Climate Change and Extreme Weather Events–Implications for Food Production, Plant Diseases, and Pests. Glob. Chang..

[12] Deikman J., Petracek M., Heard J.E. (2012). Drought Tolerance through Biotechnology: Improving Translation from the Laboratory to Farmers’ Fields. Curr. Opin. Biotechnol..

[13] Umezawa T., Fujita M., Fujita Y., Yamaguchi-Shinozaki K., Shinozaki K. (2006). Engineering Drought Tolerance in Plants: Discovering and Tailoring Genes to Unlock the Future. Curr. Opin. Biotechnol..

[14] Yang S., Vanderbeld B., Wan J., Huang Y. (2010). Narrowing Down the Targets: Towards Successful Genetic Engineering of Drought-Tolerant Crops. Mol. Plant.

[15] Guo M., Rupe M.A., Wei J., Winkler C., Goncalves-Butruille M., Weers B.P., Cerwick S.F., Dieter J.A., Duncan K.E., Howard R.J. (2014). Maize ARGOS1 (ZAR1) Transgenic Alleles Increase Hybrid Maize Yield. J. Exp. Bot..

[16] Nelson D.E., Repetti P.P., Adams T.R., Creelman R.A., Wu J., Warner D.C., Anstrom D.C., Bensen R.J., Castiglioni P.P., Donnarummo M.G. (2007). Plant Nuclear Factor Y (NF-Y) B Subunits Confer Drought Tolerance and Lead to Improved Corn Yields on Water-Limited Acres. Proc. Natl. Acad. Sci. USA.

[17] Habben J.E., Bao X., Bate N.J., DeBruin J.L., Dolan D., Hasegawa D., Helentjaris T.G., Lafitte R.H., Lovan N., Mo H. (2014). Transgenic Alteration of Ethylene Biosynthesis Increases Grain Yield in Maize under Field Drought-Stress Conditions. Plant Biotechnol. J..

[18] Li B., Wei A., Song C., Li N., Zhang J. (2008). Heterologous Expression of the TsVP Gene Improves the Drought Resistance of Maize. Plant Biotechnol. J..

[19] Nuccio M.L., Wu J., Mowers R., Zhou H.-P., Meghji M., Primavesi L.F., Paul M.J., Chen X., Gao Y., Haque E. (2015). Expression of Trehalose-6-Phosphate Phosphatase in Maize Ears Improves Yield in Well-Watered and Drought Conditions. Nat. Biotechnol..

[20] Quan R., Shang M., Zhang H., Zhao Y., Zhang J. (2004). Engineering of Enhanced Glycine Betaine Synthesis Improves Drought Tolerance in Maize: Glycine Betaine Improves Maize Drought Tolerance. Plant Biotechnol. J..

[21] Shou H. (2004). Expression of the Nicotiana Protein Kinase (NPK1) Enhanced Drought Tolerance in Transgenic Maize. J. Exp. Bot..

[22] Nguyen T.X. (2013). Barley HVA1 Gene Confers Drought and Salt Tolerance in Transgenic Maize *Zea mays* L.. Adv. Crop. Sci. Tech..

[23] Zhang S., Li N., Gao F., Yang A., Zhang J. (2010). Over-Expression of TsCBF1 Gene Confers Improved Drought Tolerance in Transgenic Maize. Mol. Breed..

[24] Thannickal V.J., Fanburg B.L. (2000). Reactive Oxygen Species in Cell Signaling. Am. J. Physiol. Lung Cell. Mol. Physiol..

[25] Sofo A., Scopa A., Nuzzaci M., Vitti A. (2015). Ascorbate Peroxidase and Catalase Activities and Their Genetic Regulation in Plants Subjected to Drought and Salinity Stresses. Int. J. Mol. Sci..

[26] Mashamaite L.N., Rohwer J.M., Pillay C.S. (2015). The Glutaredoxin Mono- and Di-Thiol Mechanisms for Deglutathionylation Are Functionally Equivalent: Implications for Redox Systems Biology. Biosci. Rep..

[27] Rouhier N. (2010). Plant Glutaredoxins: Pivotal Players in Redox Biology and Iron-Sulphur Centre Assembly. New Phytol..

[28] Wu Q., Yang J., Cheng N., Hirschi K.D., White F.F., Park S. (2017). Glutaredoxins in Plant Development, Abiotic Stress Response, and Iron Homeostasis: From Model Organisms to Crops. Environ. Exp. Bot..

[29] Cheng N.-H., Liu J.-Z., Liu X., Wu Q., Thompson S.M., Lin J., Chang J., Whitham S.A., Park S., Cohen J.D. (2011). *Arabidopsis* Monothiol Glutaredoxin, AtGRXS17, Is Critical for Temperature-Dependent Postembryonic Growth and Development via Modulating Auxin Response. J. Biol. Chem..

[30] Hu Y., Wu Q., Sprague S.A., Park J., Oh M., Rajashekar C.B., Koiwa H., Nakata P.A., Cheng N., Hirschi K.D. (2015). Tomato Expressing Arabidopsis Glutaredoxin Gene AtGRXS17 Confers Tolerance to Chilling Stress via Modulating Cold Responsive Components. Hortic. Res..

[31] Wu Q., Lin J., Liu J.-Z., Wang X., Lim W., Oh M., Park J., Rajashekar C.B., Whitham S.A., Cheng N.-H. (2012). Ectopic Expression of Arabidopsis Glutaredoxin AtGRXS17 Enhances Thermotolerance in Tomato: Ectopic Expression of AtGRXS17 in Tomato. Plant Biotechnol. J..

[32] Wu Q., Hu Y., Sprague S.A., Kakeshpour T., Park J., Nakata P.A., Cheng N., Hirschi K.D., White F.F., Park S. (2017). Expression of a Monothiol Glutaredoxin, AtGRXS17, in Tomato (*Solanum lycopersicum*) Enhances Drought Tolerance. Biochem. Biophys. Res. Commun..

[33] Yu H., Yang J., Shi Y., Donelson J., Thompson S.M., Sprague S., Roshan T., Wang D.-L., Liu J., Park S. (2017). Arabidopsis Glutaredoxin S17 Contributes to Vegetative Growth, Mineral Accumulation, and Redox Balance during Iron Deficiency. Front. Plant Sci..

[34] USDA Foreign Agricultural Service (2020). World Agricultural Production. Ag Data Commons. https://data.nal.usda.gov/dataset/world-agricultural-production.

[35] Çakir R. (2004). Effect of Water Stress at Different Development Stages on Vegetative and Reproductive Growth of Corn. Field Crop. Res..

[36] Atteya A.M. (2003). Alteration of water relations and yield of corn genotypes in response to drought stress. Bulg. J. Plant Physiol..

[37] Aylor D.E. (2003). Rate of Dehydration of Corn (*Zea mays* L.) Pollen in the Air. J. Exp. Bot..

[38] Luna V.S., Figueroa M.J., Baltazar M.B., Gomez L.R., Townsend R., Schoper J.B. (2001). Maize Pollen Longevity and Distance Isolation Requirements for Effective Pollen Control. Crop Sci..

[39] Fonseca A.E., Westgate M.E. (2005). Relationship between Desiccation and Viability of Maize Pollen. Field Crop. Res..

[40] Ouyang W., Struik P.C., Yin X., Yang J. (2017). Stomatal Conductance, Mesophyll Conductance, and Transpiration Efficiency in Relation to Leaf Anatomy in Rice and Wheat Genotypes under Drought. J. Exp. Bot..

[41] Cruz de Carvalho M.H. (2008). Drought Stress and Reactive Oxygen Species: Production, Scavenging and Signaling. Plant Signal. Behav..

[42] Kar R.K. (2011). Plant Responses to Water Stress: Role of Reactive Oxygen Species. Plant Signal. Behav..

[43] Merah O. (2001). Potential Importance of Water Status Traits for Durum Wheat Improvement under Mediterranean Conditions. J. Agric. Sci..

[44] Khayatnezhad M., Gholamin R., Jamaati-e-Somarin S., Zabihi-e R. (2011). The Leaf Chlorophyll Content and Stress Resistance Relationship Considering in Corn Cultivars (*Zea. mays*). Adv. Environ. Biol..

[45] Keyvan S. (2010). The Effects of Drought Stress on Yield, Relative Water Content, Proline, Soluble Carbohydrates and Chlorophyll of Bread Wheat Cultivars. J. Anim. Plant Sci..

[46] Lichtenthaler H.K., Buschmann C. (2001). Chlorophylls and Carotenoids: Measurement and Characterization by UV-VIS Spectroscopy. Curr. Protoc. Food Anal. Chem..

[47] Ibarra-Caballero J., Villanueva-Verduzco C., Molina-Galán J., Sánchez-De-Jiménez E. (1988). Proline Accumulation as a Symptom of Drought Stress in Maize: A Tissue Differentiation Requirement. J. Exp. Bot..

[48] Verslues P.E., Kim Y.-S., Zhu J.-K. (2007). Altered ABA, Proline and Hydrogen Peroxide in an Arabidopsis Glutamate:Glyoxylate Aminotransferase Mutant. Plant Mol. Biol..

[49] Vishwakarma K., Upadhyay N., Kumar N., Yadav G., Singh J., Mishra R.K., Kumar V., Verma R., Upadhyay R.G., Pandey M. (2017). Abscisic Acid Signaling and Abiotic Stress Tolerance in Plants: A Review on Current Knowledge and Future Prospects. Front. Plant Sci..

[50] Beardsell M.F., Cohen D. (1975). Relationships between Leaf Water Status, Abscisic Acid Levels, and Stomatal Resistance in Maize and Sorghum. Plant Physiol..

[51] Wang C., Yang A., Yin H., Zhang J. (2008). Influence of Water Stress on Endogenous Hormone Contents and Cell Damage of Maize Seedlings. J. Integr. Plant Biol..

[52] Brocard I.M., Lynch T.J., Finkelstein R.R. (2002). Regulation and Role of the Arabidopsis *Abscisic Acid-Insensitive 5* Gene in Abscisic Acid, Sugar, and Stress Response. Plant Physiol..

[53] Waterland N.L., Campbell C.A., Finer J.J., Jones M.L. (2010). Abscisic Acid Application Enhances Drought Stress Tolerance in Bedding Plants. HortScience.

[54] Wei L., Wang L., Yang Y., Wang P., Guo T., Kang G. (2015). Abscisic Acid Enhances Tolerance of Wheat Seedlings to Drought and Regulates Transcript Levels of Genes Encoding Ascorbate-Glutathione Biosynthesis. Front. Plant Sci..

[55] Wang Y., Ma F., Li M., Liang D., Zou J. (2011). Physiological Responses of Kiwifruit Plants to Exogenous ABA under Drought Conditions. Plant Growth Regul..

[56] Finkelstein R.R., Lynch T.J. (2000). The Arabidopsis Abscisic Acid Response Gene ABI5 Encodes a Basic Leucine Zipper Transcription Factor. Plant Cell.

[57] Lopez-Molina L., Mongrand S., Chua N.-H. (2001). A Postgermination Developmental Arrest Checkpoint Is Mediated by Abscisic Acid and Requires the ABI5 Transcription Factor in Arabidopsis. Proc. Natl. Acad. Sci. USA.

[58] Skubacz A., Daszkowska-Golec A., Szarejko I. (2016). The Role and Regulation of ABI5 (ABA-Insensitive 5) in Plant Development, Abiotic Stress Responses and Phytohormone Crosstalk. Front. Plant Sci..

[59] Yan F., Deng W., Wang X., Yang C., Li Z. (2012). Maize (*Zea mays* L.) Homologue of ABA-Insensitive (ABI) 5 Gene Plays a Negative Regulatory Role in Abiotic Stresses Response. Plant Growth Regul..

[60] Zou M., Guan Y., Ren H., Zhang F., Chen F. (2008). A BZIP Transcription Factor, OsABI5, Is Involved in Rice Fertility and Stress Tolerance. Plant Mol. Biol..

[61] Anjum S.A., Ashraf U., Tanveer M., Khan I., Hussain S., Shahzad B., Zohaib A., Abbas F., Saleem M.F., Ali I. (2017). Drought Induced Changes in Growth, Osmolyte Accumulation and Antioxidant Metabolism of Three Maize Hybrids. Front. Plant Sci..

[62] Zhang Z., Zhang Q., Wu J., Zheng X., Zheng S., Sun X., Qiu Q., Lu T. (2013). Gene Knockout Study Reveals That Cytosolic Ascorbate Peroxidase 2(OsAPX2) Plays a Critical Role in Growth and Reproduction in Rice under Drought, Salt and Cold Stresses. PLoS ONE.

[63] Zhang J., Kirkham M.B. (1996). Antioxidant Responses to Drought in Sunflower and Sorghum Seedlings. New Phytol..

[64] Apel K., Hirt H. (2004). Reactive Oxygen Species: Metabolism, Oxidative Stress, and Signal Transduction. Annu. Rev. Plant Biol..

[65] Rouhier N., Villarejo A., Srivastava M., Gelhaye E., Keech O., Droux M., Finkemeier I., Samuelsson G., Dietz K.J., Jacquot J.-P. (2005). Identification of Plant Glutaredoxin Targets. Antioxid. Redox Signal..

[66] Priya M., Dhanker O.P., Siddique K.H.M., HanumanthaRao B., Nair R.M., Pandey S., Singh S., Varshney R.K., Prasad P.V.V., Nayyar H. (2019). Drought and Heat Stress-Related Proteins: An Update about Their Functional Relevance in Imparting Stress Tolerance in Agricultural Crops. Theor. Appl. Genet..

[67] Augustine S.M., Hossain M.A., Wani S.H., Bhattacharjee S., Burritt D.J., Tran L.-S.P. (2016). Function of Heat-Shock Proteins in Drought Tolerance Regulation of Plants. Drought Stress Tolerance in Plants, Vol 1: Physiology and Biochemistry.

[68] Song H., Zhao R., Fan P., Wang X., Chen X., Li Y. (2009). Overexpression of AtHsp90.2, AtHsp90.5 and AtHsp90.7 in Arabidopsis Thaliana Enhances Plant Sensitivity to Salt and Drought Stresses. Planta.

[69] Sun X., Zhu J., Li X., Li Z., Han L., Luo H. (2020). AsHSP26.8a, a Creeping Bentgrass Small Heat Shock Protein Integrates Different Signaling Pathways to Modulate Plant Abiotic Stress Response. BMC Plant Biol..

[70] Martins L., Knuesting J., Bariat L., Dard A., Freibert S.A., Marchand C.H., Young D., Dung N.H.T., Voth W., Debures A. (2020). Redox Modification of the Iron-Sulfur Glutaredoxin GRXS17 Activates Holdase Activity and Protects Plants from Heat Stress. Plant Physiol..

[71] Sprague S.A. (2018). Ectopic Expression of an Arabidopsis Glutaredoxin Increases Thermotolerance in Maize during Reproductive Developmental Stages. Ph.D. Thesis.

[72] Hu Y., Wu Q., Peng Z., Sprague S.A., Wang W., Park J., Akhunov E., Jagadish K.S.V., Nakata P.A., Cheng N. (2017). Silencing of OsGRXS17 in Rice Improves Drought Stress Tolerance by Modulating ROS Accumulation and Stomatal Closure. Sci. Rep..

[73] Lichtenthaler H.K. (1987). Chlorophylls and Carotenoids: Pigments of Photosynthetic Biomembranes. Methods Enzymol..

[74] Barrs H.D., Weatherley P.E. (1962). A Re-examination of the Relative Turgidity Technique for Estimating Water Deficits in Leaves. Aust. J. Biol. Sci..

