# Supplementary material for: Ectopic Expression of a Heterologous Glutaredoxin Enhances Drought Tolerance and Grain Yield in Field Grown Maize
Source: Int J Mol Sci. 2021 May 19;22(10):5331. doi: 10.3390/ijms22105331 (PMC8158702; doi:10.3390/ijms22105331)
Supplement: Supplementary file 1 [file ijms-22-05331-s001.zip › ijms-1212140-supplementary.pdf]

Supplementary Figures

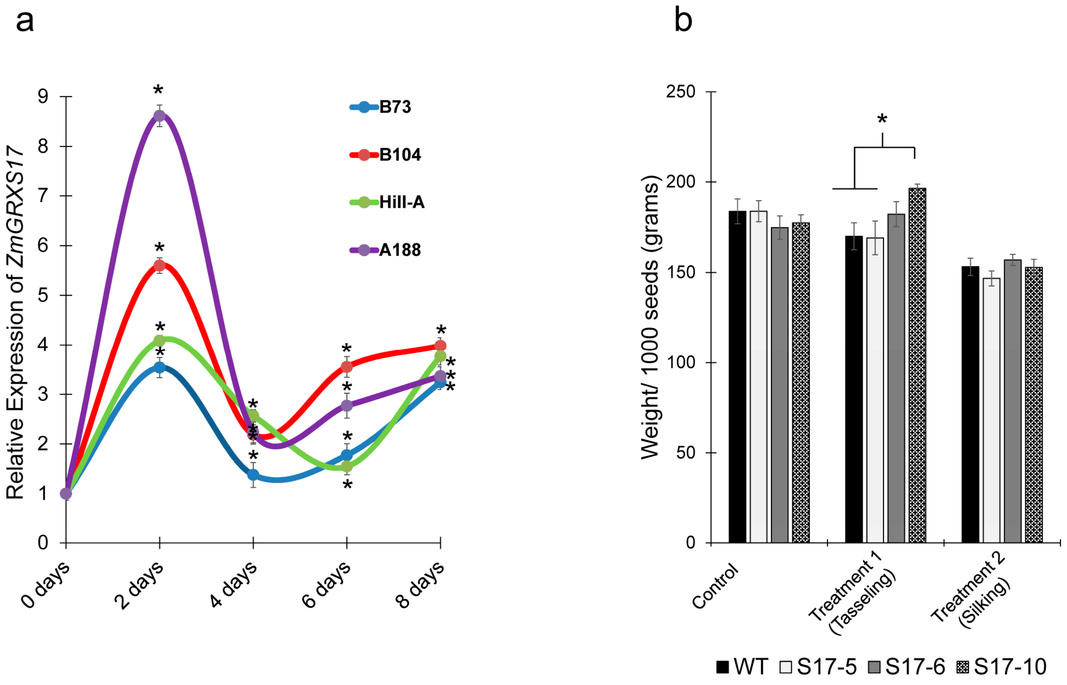

Supplementary Figure S1 Expression levels of ZmGRXS17 in inbred lines. (a) Time course normalized relative expression of ZmGRXS17 in response to drought over 8-day period in greenhouse (26°C/22°C, day/night) of B73, B104, HiII-A and A188. Leaf samples were collected at 2-day interval. Data shown are Mean ± SE (n = 3) and were analyzed using Student's t-test. Asterisks denote the difference between the corresponding time point and day 0 control (*P<0.05). (b) Weight of 1,000 seeds per plant from maize plants grown in well-watered condition and drought at the VT (n = 12) and R1 (n = 12) stages in the field. Data shown are Mean ± SE and were analyzed using one-way ANOVA. Asterisks indicate level of significance (*P<0.05).

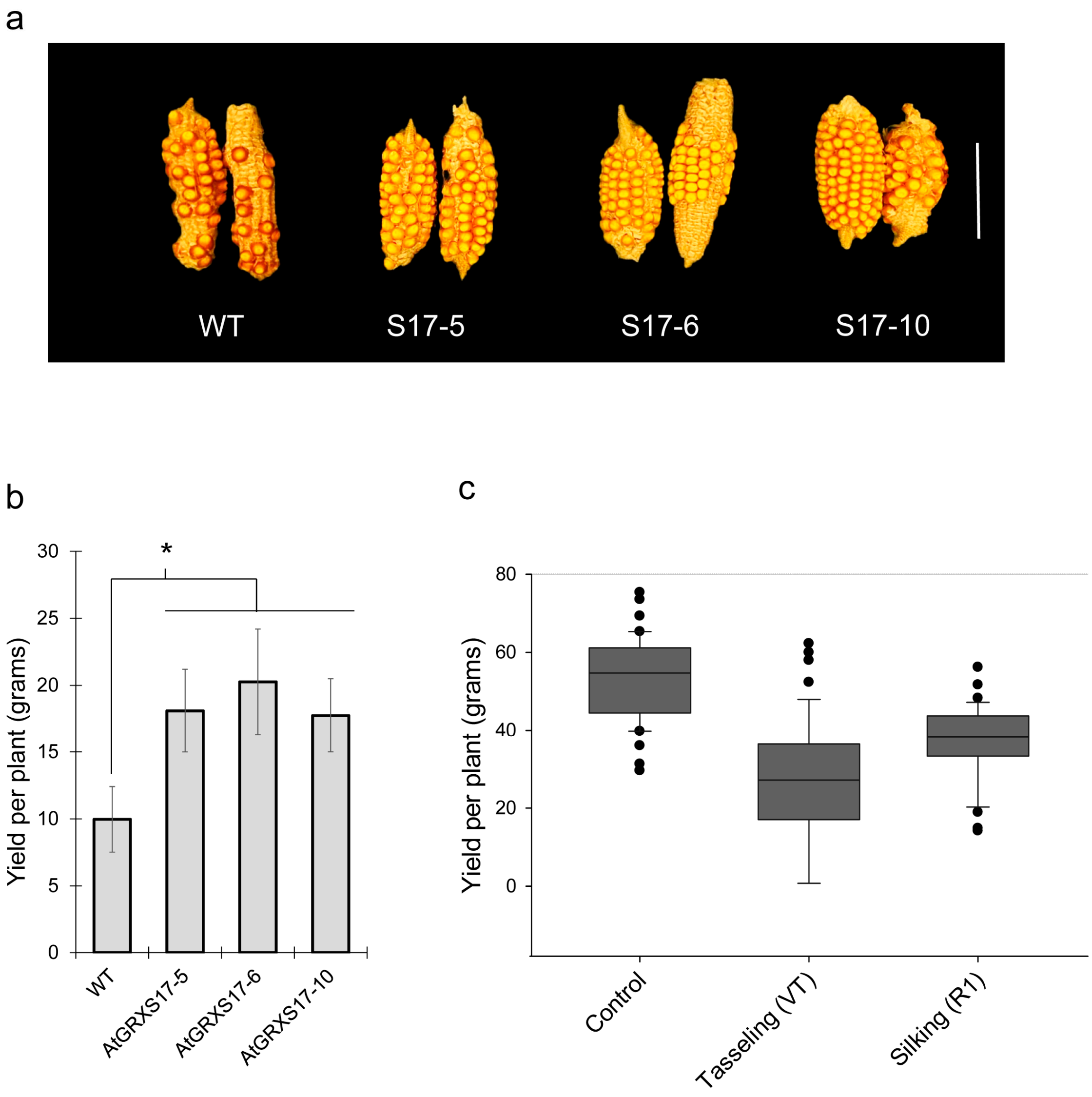

Supplementary Figure S2 Effect of *AtGRXS17* expression in maize yield under drought stress. **(a)** Representative cobs from WT and *AtGRXS17*-expressing lines grown in the greenhouse under drought condition at VT stage. Scale bar = 10 cm. **(b)** Average yield per maize plant grown under the drought stress in the greenhouse (n = 15). **(c)** Comparison of yield per plant under drought condition at VT and R1 stages. The drought had severe effect in the yield of the maize. The median yield per plant in grams for control, treatment 1 (VT) and treatment 2 (R1) were 54.7 g, 27.2 g and 38.2 g respectively. Data shown are Mean ± SE and were analyzed using one-way ANOVA. Asterisks indicate level of significance (*P<0.05).

Supplementary tables

Table S1 Primers used for qRT-PCR

Gene	Gene ID	Primers
Arabidopsis Glutaredoxin 17 (AtGRXS17)	AT4G04950	Forward: AGTCGTGCTTCACTTCTGGG<br>Reverse: CCAACCCTAAGCTTGCAGGA
Maize Glutaredoxin 17 (ZmGRXS17)	GRMZM2G131769	Forward: CCTGAGTCTGCAACTGAGAAG<br>Reverse: CATCACTGGGCTGGAGTTAAT
Catalase 1 (ZmCAT1)	GRMZM2G088212	Forward: ACAGGCTGTCGTGAGAAGTG<br>Reverse: TACGGTGTTCATGGGTCACG
L-ascorbate peroxidase (ZmAPX)	GRMZM2G156227	Forward: TAAAACAGGCCTTCCAGGGG<br>Reverse: TGCCTTTCTGGAGATCGCTG
ABSCISIC ACID-INSENSITIVE 5 (ZmABI5)	GRMZM2G479760	Forward: CGCTAAGCCACTCTACTCCG<br>Reverse: GAGAGAGTACACCGACCCCT
Brassinosteroid synthesis 1 (Zmbrs1)	GRMZM2G065635	Forward: AGGAAGATGGAGGACAGGCT<br>Reverse: CGTTTATAACCCGGAGTTCTTGC
Heat shock protein, 90 KDa (ZmHSP90)	GRMZM5G833699	Forward: TTTCGGGTCTGCTCTGAGTG<br>Reverse: CTTGGGCATCTGTTGAGCTTC
Heat Shock protein, 26 KDa (ZmHSP26)	GRMZM2G149647	Forward: TGTCCGGAAGCTTCAACGTC<br>Reverse: TTTCCTGGTTGGAGACCGTG
